# Inhibition properties of free and conjugated leupeptin analogues

**DOI:** 10.1002/2211-5463.12994

**Published:** 2020-11-02

**Authors:** Erika Billinger, Johan Viljanen, Sara Bergström Lind, Gunnar Johansson

**Affiliations:** ^1^ Department of Chemistry–BMC, Biochemistry Uppsala University Uppsala Sweden; ^2^ Bjerking AB Uppsala Uppsala Sweden; ^3^ Department of Chemistry–BMC, Organic Chemistry Uppsala University Uppsala Sweden; ^4^ Department of Chemistry–BMC, Analytical Chemistry Uppsala University Uppsala Sweden; ^5^ Office for Science and Technology Uppsala University Uppsala Sweden

**Keywords:** conjugation, inhibition, inhibitor design, leupeptin analogues, tight binding

## Abstract

Leupeptin is a naturally occurring inhibitor of various proteases, in particular serine proteases. Following its discovery, the inhibitory properties of several other peptidyl argininals have been studied. The specificity of leupeptin is most likely due to the Leu–Leu–Argininal sequence, and its C‐terminal aldehyde group has been suggested to enhance the binding efficiency and to be essential for function. The terminal aldehyde group makes the structure less vulnerable to carboxypeptidases. Here, we investigated whether the inhibitory function of leupeptin toward serine proteases is retained after oxidation or reduction of the aldehyde group. The oxidized form, which corresponds to the natural precursor, was shown to be superior to the reduced form in terms of inhibitory properties. However, the original leupeptin possessed enhanced inhibitory properties as compared with the oxidized form. Based on these results, new synthetic leupeptin analogues, 6‐aminohexanoic acid (Ahx)–Phe–Leu–Arg–COOH and Ahx–Leu–Leu–Arg–COOH, were prepared by solid‐phase peptide synthesis using the Fmoc strategy. In these analogues, the N‐terminal capping acetyl group was replaced with a 6‐aminohexanoyl group to allow conjugation. The structures of the modified leupeptin and the synthetic peptides were confirmed by mass spectrometry. Determination of the inhibitory properties against trypsin (IEC 3.4.21.4, Chymotrypsin IEC 3.4.21.1) revealed that these further modified tripeptides were tight binding inhibitors to their target enzyme, similar to the naturally occurring leupeptin, with *K_i_* values generally in the micromolar range. The Ahx–Phe–Leu–Arg–COOH analogue was selected for conjugation to inorganic oxide nanoparticles and agarose gel beads. All conjugates exhibited inhibitory activity in the same range as for the free peptides.

## Graphical abstract

Here, we see the path from original leupeptin to a designed and conjugated peptide analogue, starting on top with the original peptide aldehyde. The next view represents the designed peptide, and finally, we see a representation of the working conjugate at the bottom.

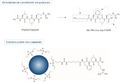

**Abbreviations**
ACN: acetonitrile
Ahx: 6‐aminohexanoic acid
APTES: aminopropyltriethoxysilane
BAPA: *N*α‐benzoyl‐dl‐arginine‐4‐nitroanilide hydrochloride
CDI: carbonyldiimidazole
MS: mass spectrometry
SPPS: solid‐phase peptide synthesis
TFA: trifluoroacetic acid

Leupeptin (Fig. 1) is known to be a naturally occurring, efficient, tight‐binding inhibitor to various proteases, in particular serine proteases [1, 2, 3, 4, 5, 6, 7, 8]. Leupeptin is produced by various species of actinomycetes and was also confirmed in a number of other families. The production is therefore not species specific [9]. Since the discovery of leupeptin, several additional peptidyl argininals have been subject to interest because of the strong and specific inhibition of serine proteases [10]. The specificity of leupeptin is most likely due to the Leu–Leu–Argininal sequence, and the C‐terminal aldehyde function is suggested to enhance the binding efficiency and to be essential for the function [1, 11, 12]. It is also likely that the terminal aldehyde group will make the structure less vulnerable to carboxypeptidases. Aoyagi *et al*. [2] reported that the reduction or oxidation of the aldehyde group to alcohol or carboxylate, respectively, strongly impaired the inhibition of plasmin and papain, to the extent that no exact numerical data were given. Our aim here was to investigate whether the inhibitory function toward serine proteases is retained also after oxidation or reduction of the aldehyde group. The biosynthesis of leupeptin does indeed occur via a synthesis of the peptide with a C‐terminal carboxyl group, followed by a reduction to aldehyde catalyzed by the enzyme leupeptin acid reductase with NADH as electron donor and thermodynamically supported by ATP hydrolysis [13], in analogy with that the chemical synthesis of leupeptin is thus more complicated than standard peptide synthesis due to its l‐argininal moiety. Many analogues to leupeptin have nevertheless been synthesized, giving a broad view of structure–function relations for the inhibition of proteases [12, 14, 15].

**Fig. 1:**
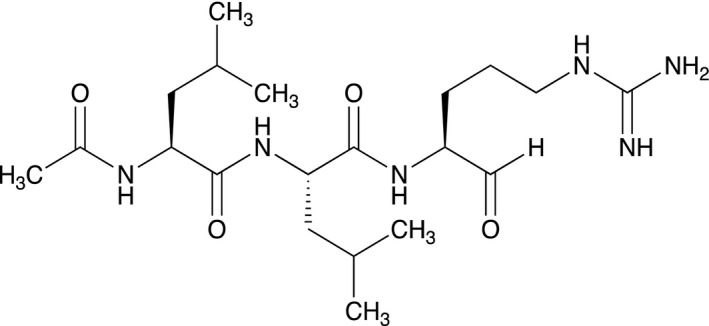
Leupeptin in its natural state (Ac–Leu–Leu–Arg–CHO).

Here we are using solid‐phase peptide synthesis (SPPS) to synthesize two new leupeptin analogues with an ‐COOH at the C terminus instead of an aldehyde, similar to the biosynthetic precursor. Staying at the precursor stage does thus allow a straightforward SPPS. One option to synthesize leupeptin to its natural state could actually be based on an enzymatic reduction after the SPPS of the oxidized form. As of today, no enzyme that catalyzes the reduction of the C‐terminal carboxyl group of the precursor to aldehyde in *Streptomyces* is commercially available [13]. The N‐terminal acetyl group was in these tripeptide analogues of leupeptin replaced with a 6‐aminohexanoyl group that allows an easy path to conjugation while retaining inhibitory activity. The nucleophilic character of amino groups is frequently used in the conjugation of peptides, amino acids and proteins, and a large number of activations of conjugation carriers have been developed [14, 15, 16]. The gel support used in this study was the bromo‐activated WorkBeads™ matrix, where the conjugation results in a secondary amine. For conjugation to inorganic oxide phases, an initial silanization is often used to introduce suitable functional groups. In this case, we have chosen to modify the oxide surfaces with aminopropyltriethoxysilane (APTES), followed by activation of the amino groups by carbonyldiimidazole (CDI). The final result will be a coupling of the peptides by a substituted urea structure. Because the peptides carry only a single primary amino group, the conjugation is in all cases expected to be uniform and, furthermore, leave the important arginine accessible.

## Results and Discussion

### Oxidation and reduction of leupeptin

**Fig. 2:**
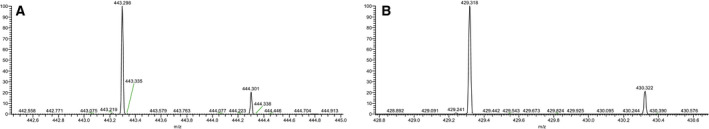
(A) MS spectra of oxidized leupeptin with a peak at 443 *m/z* corresponding to the molecular ion [M + H]. (B) MS spectra of reduced leupeptin with a peak at 429 *m/z* corresponding to the molecular ion [M + H], confirming the identity of both products.

The molecular weight of the oxidized and reduced state of leupeptin was determined by mass spectrometry (MS) analysis and can be seen in Fig. 2, where the oxidized state shows a peak at 443 *m/z* and the reduced state shows a peak at 429 *m/z*, with *z* = 1 for both peptides. This is in expected accordance with the peak at 427 *m/z* for natural leupeptin (Table 1). Full *m/z* spectra can be found in Fig. S1A,B.

**Table 1:** Summary of the representative results from the reduction and oxidation of leupeptin, including the obtained kinetic parameters for the different state of the inhibitors.

	State	Functional group	*K_i_*	*m/z* observed	*z*	*M*_w_ (Da)
Leupeptin	Natural	‐CHO	88 ± 8 nm	427.3	+1	426.3
	Oxidized	‐COOH	2.7 ± 0.1 μm	443.3	+1	442.3
	Reduced	‐CH_2_OH	270 ± 100 μm	429.3	+1	428.3

### Inhibitor binding mode

The original natural leupeptin interacts very strongly but reversibly with trypsin (IEC 3.4.21.4, Chymotrypsin IEC 3.4.21.1). The backbone of leupeptin forms four hydrogen bonds with trypsin, and a fifth hydrogen bond interaction is mediated by a water molecule. The aldehyde carbonyl of leupeptin is shown to form a hemiacetal bond with the side‐chain oxygen of Ser195 in the active site, in which the hemiacetal oxygen atom is pointing out of the oxyanion hole and forms a hydrogen bond with His57 [17]. Radisky *et al*. [18] modeled both orientations of the hemiacetal and found that the one where the oxygen atom is facing the oxyanion hole had 15% occupancy, whereas the one where oxygen faces the active site His57 had 85% occupancy.

**Fig. 3:**
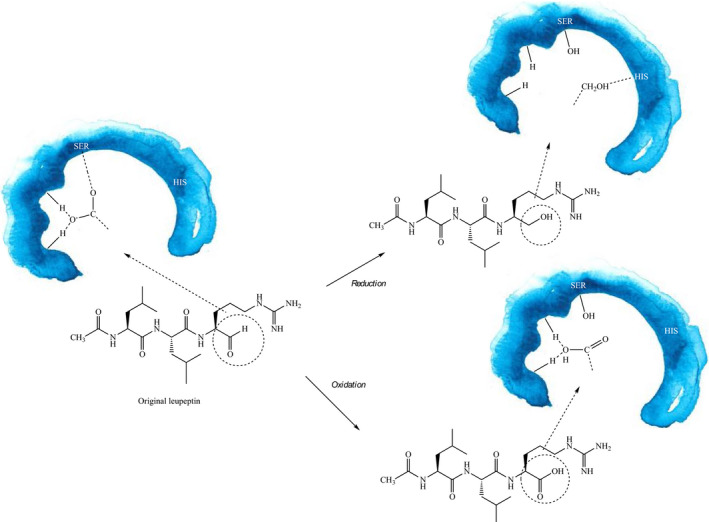
Tentative comparison of active site binding mode for original, reduced and oxidized leupeptin, respectively.

The weaker inhibition by both the oxidized and reduced state of leupeptin can be explained by the loss of the hemiacetal bond possibility in the active site to Ser195. Tentative binding modes for original and modified leupeptin are shown in Fig. 3. The stronger inhibition by the oxidized form compared with the reduced form can be explained by the interaction of the negative oxygen in the oxyanion hole, but without the hemiacetal formation. The reduced state of leupeptin does also lack the possibility to form a hemiacetal but can still interact through a hydrogen bond to His57. This could be the reason for the pronounced difference between an apparent *K_i_* of 2.69 μm for the oxidized form and 270 μm for the reduced form (Table 1). Based on these data, we decided to synthesize analogues with the terminal carboxyl group.

### Synthesis of 6‐aminohexanoic acid–Phe–Leu–COOH and 6‐aminohexanoic acid–Leu–Leu–COOH

The synthetic pathway of a serine protease inhibitor of leupeptin type is a challenge due to the argininal residue at the C terminus of their sequence combined with a low yield as an outcome [19]. The standard arginine peptide, on the contrary, requires only straightforward peptide synthesis and is still a powerful inhibitor. It should also be noted that the biosynthesis proceeds via synthesis of the peptide, followed by a specific enzyme‐mediated reduction of the terminal carboxylate group to aldehyde [13]. Conclusively, chemical synthesis of a precursor peptide combined with a final enzymatic reduction could actually be the preferred strategy for production of leupeptin analogues with a terminal aldehyde group.

#### Purification and characterization of 6‐aminohexanoic acid–Phe–Leu–Arg–COOH and 6‐aminohexanoic acid–Leu–Leu–Arg–COOH

The two peptides, 6‐aminohexanoic acid (Ahx)–Phe–Leu–Arg–COOH and Ahx–Leu–Leu–Arg–COOH, were purified with preparative HPLC. The yields of the peptides were 36% and 74%, respectively, based on the capacity of the resin. The *m/z* relation was 514 with *z* = 1 for Ahx–Leu–Leu–Arg–COOH and 548.4 with *z* = 1 for Ahx–Phe–Leu–Arg–COOH by liquid chromatography MS, which confirmed the molecular masses of the peptides to 513 Da for Ahx–Leu–Leu–Arg–COOH and to 547 Da for Ahx–Phe–Leu–Arg–COOH.

#### Kinetic measurements of Ahx–Phe–Leu–Arg–COOH and Ahx–Leu–Leu–Arg–COOH

**Fig. 4:**
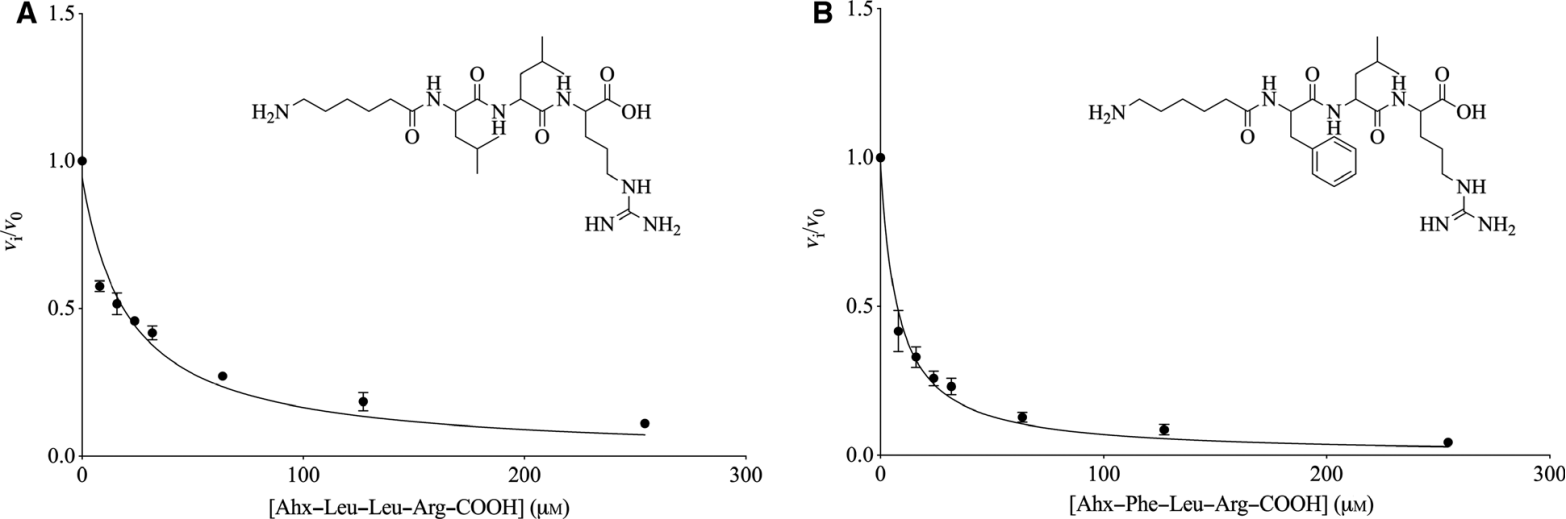
The ratio *v_i_*/*v*_0_ is plotted as a function of the inhibitor peptide Ahx–Phe–Leu–Arg–COOH (A) and Ahx–Leu–Leu–Arg–COOH (B). The conditions for the experiments and calculations were [trypsin] = 0.25 μm, [BAPA] = 1 mm and *K*_m_ = 0.82 mm. Error bars represent standard deviation.

The apparent *K_i_* values of Ahx–Leu–Leu–Arg–COOH (9.48 μm) and Ahx–Phe–Leu–Arg–COOH (3.42 μm) lie in the same micromolar range as oxidized leupeptin (Tables 1 and 3), confirming that the replacement of the N‐terminal capping acetyl group with AHX has only a minor influence. The replacement of one leucine by phenylalanine does, furthermore, lower the *K_i_* value by a factor 3. This is probably due to the fact that phenylalanine is more hydrophobic than leucine and interacts more strongly in the active site of the enzyme. Both the synthesized peptides act as a tight binding inhibitor, as shown in Fig. 4. Even though the hemiacetal function is lost by synthesizing them with a carboxylic acid instead of the functional aldehyde, these peptides still show significant inhibition properties.

### Conjugated Ahx–Phe–Leu–Arg–COOH

#### Conjugation of Ahx–Phe–Leu–Arg–COOH

Ahx–Phe–Leu–Arg–COOH was conjugated to three different carriers where the conjugated amount was determined by subtractive absorbance measurements and is shown in Table 2.

**Table 2:** Summary of the amount of Ahx–Phe–Leu–Arg–COOH immobilized onto the chosen carriers.

Alternative	Carriers	Conjugated amount of Ahx–Phe–Leu–Arg–COOH
Inorganic particles	TiO_2_	6.1 nmol·mg^−1^ particle
	ZnO	4.4 nmol·mg^−1^ particle
Gel beads	WorkBeads™	150 nmol·mL^−1^ beads

#### Kinetic measurements of conjugated Ahx–Phe–Leu–Arg–COOH

**Fig. 5:**
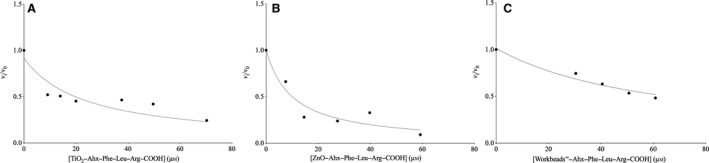
*v_i_*/*v*_0_ is plotted as a function of the conjugated TiO_2_–Ahx–Phe–Leu–Arg–COOH (A), ZnO–Ahx–Phe–Leu–Arg–COOH (B) and WorkBeads™‐Ahx‐Phe‐Leu‐Arg‐COOH (C). The conditions for the experiments and calculations were [trypsin] = 0.25 μm, [BAPA] = 1 mm and *K*_m_ = 0.82 mm. Error bars represent standard deviation.

Because the synthetic analogues display the same inhibition mechanisms as the natural leupeptin (tight binding), the apparent *K_i_* values (Table 3) were determined by Eqns (1,2). The corresponding graphs can be seen later (Fig. 5). For details regarding TiO_2_‐peptide kinetics, see Fig. S2 and Table S1.

**Table 3:** Summary of the apparent *K_i_* values from the different states of inhibitors and the efficiency where free Ahx–Phe–Leu–Arg–COOH is used as the reference.

State	Carrier	Peptide	*K_i_*	Efficiency	*K_i_* stored
Free	–	Leupeptin	88 ± 8 nm	–	
		Leupeptin–COOH	2.7 ± 0.1 μm	–	
		Ahx–Leu–Leu–Arg–COOH	9.5 ± 1.0 μm	–	
		Ahx–Phe–Leu–Arg–COOH	3.4 ± 0.2 μm	1.00^a^	
Conjugated	TiO_2_	Ahx–Phe–Leu– Arg–COOH	11 ± 2 μm	0.32	7.1 ± 1.1 μm
	ZnO		4.4 ± 1.3 μm	0.77	7.6 ± 1.5 μm
	WorkBeads™		29 ± 4 μm	0.12	53 ± 9 μm

^a^ Reference value.

The increase in apparent *K_i_* can be ascribed to sterical inaccessibility as a result of an uneven surface in all three carriers. The peptides can also be conjugated close to each other, making the space for several enzyme molecules less accessible. Regarding the gel beads, the peptides can be hidden inside the beads, which will exclude a number of peptides that are conjugated to those positions and thereby not accessible for the enzymes. The cross‐linking that was observed for conjugated protein inhibitors [20] is not possible for this peptide because it has only one easily reacting group for the conjugation to take place, which, furthermore, is placed opposite to the functional group that is crucial for the inhibition. If the peptide is conjugated in a way that is not sterically inaccessible, the inhibition will not be affected by the conjugation. The calculations regarding the efficiency are based on the free Ahx–Phe–Leu–Arg–COOH as a reference, because that is the peptide that is conjugated to all three carriers. The efficiency is easiest regarded as the fraction of inhibitor molecules that are functionally active, rather than a change of intrinsic molecular properties [20], and the loss of efficiency can primarily be ascribed sterical inaccessibility. The efficiency is thus calculated from the apparent *K_i_* observed as $\frac{K_{i,\text{free}}}{K_{i,\text{app}}}\;=\;\frac{{\left[I\right]}_{\text{eff}}}{{\left[I\right]}_{\text{tot}}}$. The conjugation of the peptide is performed not to increase the stability but to merely provide a carrier to the small peptide. This may serve to remove the inhibitor in a controlled way from a technical sample but also to minimize the risk of the peptide crossing, for example, the skin barrier if used in protective formulas. As can be seen, in regard to the efficiency, the conjugation of Ahx–Phe–Leu–Arg–COOH to ZnO gives an efficiency of 0.77, which is close to the reference value of 1. The conjugation itself does not affect the peptide’s inhibition properties. The lower efficiency of the peptide conjugated to agarose gel beads could be explained by the steric inaccessibility for the enzyme, because of the possibility of the peptide hidden inside the gel beads. According to the efficiency, about 10% of the conjugated peptides are available for the enzyme.

### Inhibition activity analysis of free and conjugate peptide in gelatin layer

**Fig. 6:**
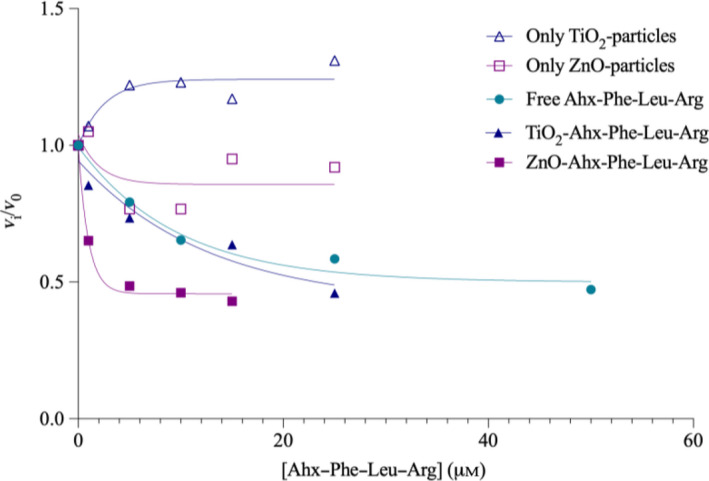
Relative area increase rate for free peptide 1, conjugated peptide 1 and free particles.

The gelatin erosion method that simulates a surface with a protective cover layer is here used to evaluate the function of the peptide in its different conjugation states where the relative area increase rate is plotted against the increasing inhibitor concentration in Fig. 6. The curves obtained as a function of concentration for the free peptide and the TiO_2_‐conjugated peptide, respectively, are virtually identical. The data obtained for the peptide–ZnO conjugate, in contrast, result in a notably steeper slope at lower inhibitor concentration, suggesting a stronger binding. This may tentatively be explained by a model where the enzyme molecules, once ‘captured’ by the immobilized peptides, also interact nonspecifically with the ZnO surface, resulting in addition of binding energies and thus also cooperative binding. As has been noted earlier by us [21], the well area converges to an asymptotic nonzero value ‘plateau’ at high inhibitor concentration that is virtually identical for the different cases. The limit may actually be set by the size of the droplet applied and initial enzyme diffusion. However, the similar limiting values further confirm that the inhibition modes are identical for free and conjugated inhibitor. The control experiments carried out with nonconjugated oxide particles resulted in plateau values close to unity, compared with the value of 0.42–0.50 for the inhibitor formulations (Table 4). The plateau value of ZnO particles decreases, however, to 0.86, suggesting a certain inhibitory effect by the oxide itself. This is actually in accordance with the pattern observed for the ZnO conjugate. Taken together, the patterns, for both slope and plateau, confirm that the conjugation does not influence the peptide function. For details regarding the gelatin erosion method, see Fig. S3 and Table S2.

**Table 4:** The relative asymptotic plateau values extracted from graphpad prism 8, where the resulting value shows the asymptotic area ratio obtained at virtually maximal inhibition.

State of peptide 1	Plateau
Free Ahx–Phe–Leu–Arg–COOH	0.50 ± 0.04
Only ZnO particles	0.86 ± 0.07
ZnO–Ahx–Phe–Leu–Arg–COOH	0.46 ± 0.02
Only TiO_2_ particles	1.24 ± 0.04
TiO_2_–Ahx–Phe–Leu–Arg–COOH	0.42 ± 0.21

## Conclusions

After background experiments where the aldehyde group of leupeptin was converted to carboxylate or alcohol group, respectively, we could design two new leupeptin analogue protease inhibitors by the use of SPPS. This design of peptides, using SPPS, is useful to create smaller peptides with inhibitory effect in a straightforward and easy synthesis. The two new peptides had a new functional group, ‐COOH, instead of the original aldehyde group of the natural state leupeptin. The N‐terminal acetyl group was also replaced by the commonly used conjugation spacer Ahx as an extra handle in the other end of the peptide for an easier conjugation path. With these modifications the peptides gave an apparent *K_i_* value in the micromolar range. The conjugation of Ahx–Phe–Leu–Arg–COOH to inorganic particles and agarose gel beads gave a retained apparent *K_i_* value in the micromolar range for all conjugates. The conclusions from these data are, first, that the leupeptin analogues still are functional inhibitors, with a more retained function for the oxidized form. Furthermore, the replacement of the original N‐terminal acetyl group with Ahx did not have a large impact on the inhibitory properties but allowed well‐defined and sterically favorable conjugation of the peptide. The data further show that the properties are retained when the peptide is immobilized to a soluble or particulate carrier. It is possible that the peptide efficiency may be further improved by application of the natural enzyme‐aided maturation reaction of natural leupeptin to generate the aldehyde function. As a consequence, and in a more general context, peptide inhibitors that are designed for conjugation may facilitate the use of their inhibitory properties both in medical formulations and as enzyme scavengers in biotechnical applications. In the latter case, it allows easy removal of conjugated inhibitors from the sample by filtering or centrifugation. The highly developed technology for peptide synthesis does also allow a both rational and virtually unlimited combinatorial approach far beyond canonical amino acids for the design of inhibitors.

## Materials and methods

### Materials

Leupeptin (L2884), AgNO_3_, sodium borohydride, *N*α‐benzoyl‐dl‐arginine‐4‐nitroanilide hydrochloride (BAPA), trypsin from porcine pancreas type IX‐S, titanium(IV) oxide, zinc oxide, 1,1ʹ‐CDI, triethylamine, APTES, DMSO, acetonitrile (ACN), formic acid, acetic acid, O‐(1H‐6‐chlorobenzotriazole‐1‐yl)‐1,1,3,3‐tetramethyluronium‐hexafluorophosphate, *N,N*‐diisopropylethylamine, dichloromethane, piperidine, acetic anhydride, trifluoroacetic acid (TFA), triisopropylsilane, Fmoc–Leu–OH, Fmoc–Phe–OH and Boc–Ahx–OH were all purchased from Sigma‐Aldrich. WorkBeads™ 40/1000 ACT was a kind gift from Bio‐Works Sweden AB, Uppsala.

### Methods

#### Oxidation of leupeptin using Tollen’s test and confirmation of reaction with MS

**Fig. 7:**
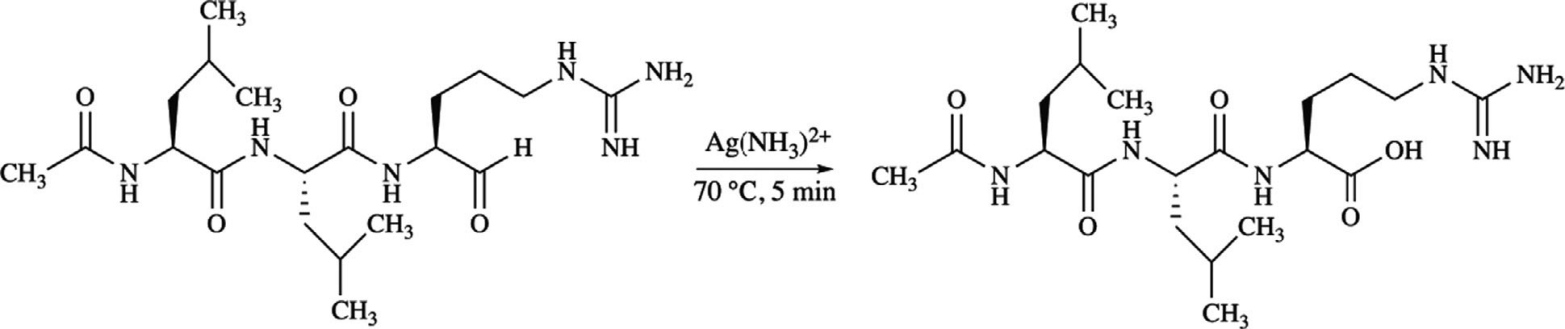
The oxidation of leupeptin using Tollen’s test.

A total of 0.25 g AgNO_3_ was dissolved in 15 mL milliQ H_2_O; 0.5 mL 1 m NaOH was added, creating a brown solution, which precipitated. The addition of 0.5 mL of 25% NH_3_ (≈ 14 m) resulted in a clear solution containing Ag(NH_3_)_2_^+^. The function of the solution was confirmed by reaction with benzaldehyde. One hundred microlitres of 5.3 mm leupeptin was mixed with 0.5 mL Ag(NH_3_)_2_^+^ incubated in a water bath (70°) for 5 min. The oxidized inhibitor (Fig. 7) was desalted by chromatography on ISOLUTE® SPE C18. Peptides (625 nm) were dissolved in 50% ACN 0.1% formic acid and directly infused by a syringe pump (Harvard Apparatur, Holliston, MA, USA) at a flow rate of 4 μL·min^−1^. Infused peptides were ionized by electrospray using an Ion Max Source (Thermo Fisher Scientific, Bremen, Germany). Peptide mass spectra were recorded over a period of 5 min, resulting in >570 spectra per peptide with a LTQ Orbitrap Velos Pro (Thermo Fisher Scientific) in the *m/z* 150–2000 range using the Orbitrap (Fourier transform) analyzer set to 30 000 resolution. The ionization potential was +4.2 kV, no sheath gas flow was used and the inlet capillary temperature was set to 300 °C. Instrument calibration was carried out according to standard operating procedures using Pierce™ LTQ Velos ESI Positive Ion Calibration Solution (Thermo Fisher Scientific) to assure high mass accuracy.

#### Reduction of leupeptin and confirmation of reaction with MS

**Fig. 8:**
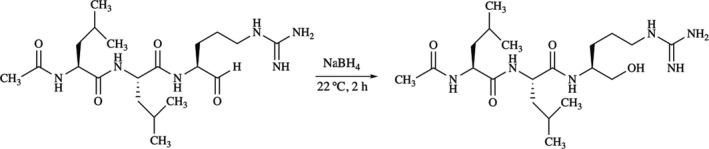
The reduction of leupeptin using sodium borohydride.

A 100‐fold molar excess of NaBH_4_ was added to a solution of 0.53 μm leupeptin dissolved in milliQ‐H_2_O. The reduction (Fig. 8) took place over a period of 2 h, followed by desalting with SPE C‐18 and was confirmed with MS detection using the same protocol as in ‘Oxidation of leupeptin using Tollen’s test and confirmation of reaction with MS’.

### Synthesis of Ahx–Phe–Leu–Arg–COOH and Ahx–Leu–Leu–Arg–COOH

#### SPPS and purification

The peptides were synthesized using Fmoc chemistry on a 50‐μmol scale, starting from Fmoc–Arg(Pbf) Wang resin (70 mg, 0.7 mmol·g^−1^). Deprotection of the Fmoc groups was carried out using 20% piperidine in dimethyl formamide. Amino acid couplings were performed in dimethyl formamide using amino acid : O‐(1H‐6‐chlorobenzotriazole‐1‐yl)‐1,1,3,3‐tetramethyluronium‐hexafluorophosphate : *N,N*‐diisopropylethylamine (5 : 4 : 10), with coupling times of 30 min to 1 h. Peptides were quantified using Kaiser’s test [22]. Total deprotection and simultaneous cleavage from the solid support was achieved with a mix of TFA/triisopropylsilane/H_2_O (95%/2.5%/2.5%, v/v, 5 mL) for 1.5 h with agitation. After filtration and evaporation of TFA by N_2_(g) bubbling, the peptides were precipitated and washed once with cold diethyl ether (Et_2_O), followed by lyophilization. For details, see the Supporting Information.

**Fig. 9:**
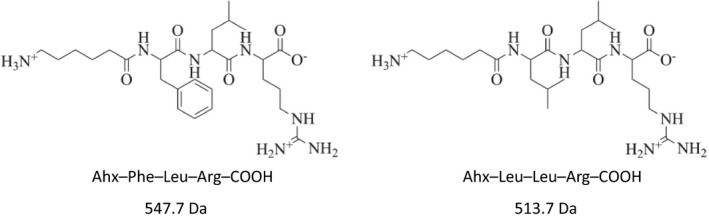
Structure of the two tripeptides.

The crude peptide was purified with reverse‐phase HPLC (Varian 940‐LC) using a semipreparative Grace Vydac C8‐column (22 mm × 150 mm, 10 µm, 300 Å) with a gradient of ACN:H_2_O, containing 0.1% TFA, from 5% (2 min) to 40% ACN in 20 min at a flow rate of 15 mL·min^−1^. UV detection was done at 220 nm. The peptide identities were confirmed by liquid chromatography MS using an instrument constellation consisting of a Waters 2700 sample manager, an Agilent 1100 series HPLC/UV‐VIS diode‐array detector, and Waters Micromass ZQ mass detector in negative ion mode. The measured *m/z* values (546.3 and 512.3, respectively) were in accordance with the calculated masses (Fig. 4). Fractions containing the desired peptides (Fig. 9) were pooled, evaporated, freeze dried and stored at −20 °C until further use.

### Conjugation of Ahx–Phe–Leu–COOH

#### Inorganic particle carriers

**Fig. 10:**
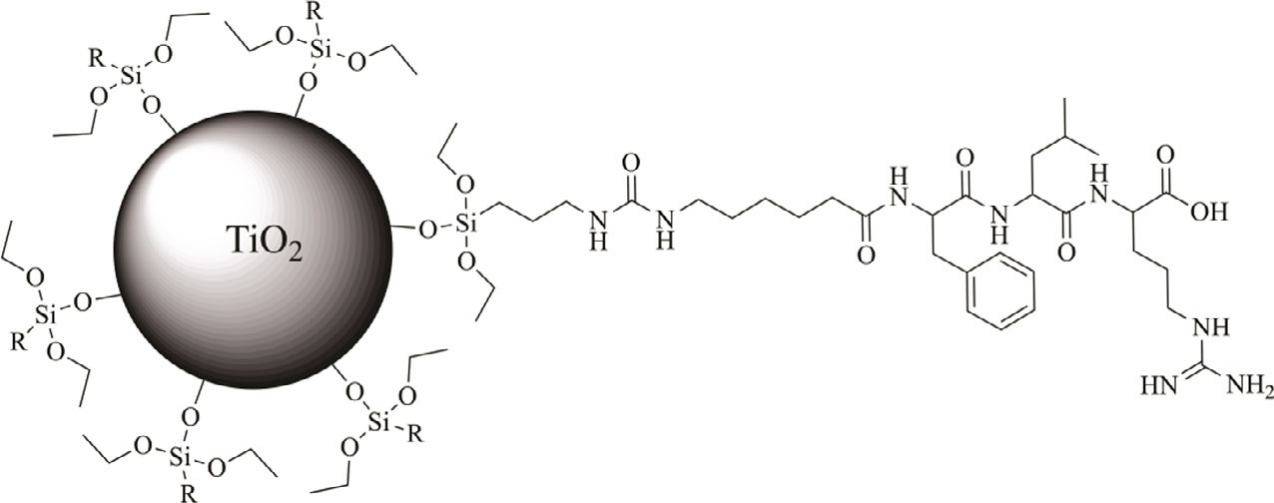
Conjugate of Ahx–Phe–Leu–Arg–COOH to TiO_2_ particles, where R = (CH_2_)_3_NHC(=O)–Ahx–Phe–Leu–Arg–COOH.

All derivatization and immobilization steps were performed at room temperature and in plastic Falcon tubes or 2‐mL plastic Eppendorf tubes. A total of 0.5 g of the inorganic particulate carriers (TiO_2_ and ZnO) was first silanized using 100 mm APTES in 10 mL ACN while stirring for 24 h. The particles were then centrifuged followed by removal of ACN, washed three times with EtOH, one final time with acetone and then dried at 65 °C for 5 h. Following that, the particles were activated using 120 mg CDI and 0.72 mmol triethylamine in 5 mL ACN while stirring for 2 h. The particles were then centrifuged followed by removal of ACN, washed three times with EtOH, one final time with acetone and then dried at 65 °C o/n. Conjugation of Ahx–Phe–Leu–Arg–COOH was carried out by stirring a controlled amount of peptide overnight with 50 mg activated TiO_2_ or ZnO in 0.1 m NH_4_HCO_3_ buffer (pH 7.52). The particles were then centrifuged, and the absorbance of the supernatants was measured at 259 nm, allowing a subtractive quantification of conjugated peptide using the extinction coefficient of 195 m ^−1^·cm^−1^ for Phe residues [23]. The conjugation was terminated by washing with 0.1 m NH_4_HCO_3_ (pH 7.52), and the conjugated particles (Fig. 10) were stored at 4 °C until further use.

#### Agarose gel beads

**Fig. 11:**
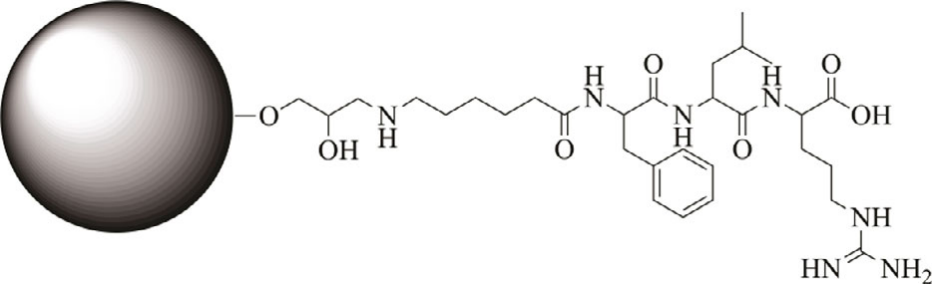
Conjugate of agarose gel bead and peptide 1. The gel bead in comparison with the peptide is about 40 000 times larger, and the gel bead can conjugate a number of peptides. This figure demonstrates only the conjugation between the bead and the peptide itself, and the components are not in correct scale.

WorkBeads™ 40/1000 ACT from Bio‐Works with an average particle size of 45 µm and reactive group content of 200 µmol·mL^−1^ were used when coupling Ahx–Phe–Leu–Arg–COOH to agarose gel beads (resin). A total of 1 mL resin was washed with deionized water on a glass filter and dried using suction. A total of 0.5 mL of 4.38 mm peptide 1 in 0.1 m NH_4_HCO_3_ (pH 7.52) was then added to 0.5 mL dried resin. The slurry mixture was incubated at room temperature overnight while stirring. Following that, the slurry mixture was centrifuged, and absorbance was measured on the supernatant. The amount conjugated was determined as mentioned earlier in ‘Inorganic particle carriers’. The immobilization was terminated by first washing the resin with 0.1 m NH_4_HCO_3_ (pH 7.52) and drying by suction followed by incubation of the resin in 1 m ethanolamine–HCl (pH 9.5) at room temperature overnight while stirring to block the remaining reactive groups. The blocking agent was finally removed by washing the gel beads as described earlier. The suspension of the conjugate (Fig. 11) was stored at 4 °C in 0.1 m NH_4_HCO_3_ (pH 7.52) until further use.

### Kinetics

#### Real‐time measurements

Kinetic parameters were obtained by measuring initial velocities in the presence of selected concentrations of natural leupeptin (0.14–1.62 µm), oxidized leupeptin (8–127 µm), reduced leupeptin (10–413 µm) and peptides 1 and 2 (8–254 µm). All reactions were performed in 0.1 m NH_4_HCO_3_ (pH 7.52) and 2% (v/v) DMSO using concentrations of BAPA and trypsin fixed at 1 mm and 0.25 µm, respectively. The reaction was monitored at 410 nm in a Shimadzu UV‐1601 UV‐Vis spectrophotometer.

#### End point measurements

Kinetic parameters were obtained by mixing substrate, enzyme and conjugate in a plastic Eppendorf tube with varying concentrations of TiO_2_–peptide 1 0–70 μm, ZnO–peptide 1 0–60 μm and gel beads conjugated to peptide 1 0–60 μm and incubating with end‐over‐end rotation. A total of 150 µL aliquots was withdrawn at five time points for each reaction, and the reaction was stopped in 100 mm acetic acid (pH 3). Absorbance was measured at 410 nm in a UV‐1601 UV‐Vis spectrophotometer (Shimadzu, Columbia, MD, USA).

For real‐time and end point measurements, the *K_i_* value was estimated by the graphical method of Dixon [24] and Morrison [25] (Eqn 1). *K_i_* was extracted after fitting the data to Eqns (1,2) using graphpad prism 8 (GraphPad Software, San Diego, CA, USA). A *K*_M_ of 0.82 mm was used for the calculations.

(1) $$\frac{v_{i}}{v_{o}}\;=\;1‐\frac{\left(\left[E\right]\;+\;\left[I\right]\;+\;K_{i}^{\text{app}}\right)\;‐\;\sqrt{{\left(\left[E\right]\;+\;\left[I\right]\;+\;K_{i}^{\text{app}}\right)}^{2}\;‐\;4\;\;\left[E\right]\;\left[I\right]}}{2\;[E]}$$

The form of $K_{\text{app}}^{i}$ varies with the type of inhibitor. For competitive inhibitors, as in the case of leupeptin, the *K_i_* value can be calculated from Eqn (2):

(2) $$K_{i}^{\text{app}}\;=\;K_{i}\;\left(1\;+\;\frac{\left[S\right]}{K_{M}}\right)$$

#### Inhibition activity analysis of free and conjugate peptide in gelatin layer

The experiments were performed as described earlier [21]. The concentration of the free inhibitor Ahx–Phe–Leu–Arg–COOH was ranging from 5 to 50 μm, for ZnO conjugate 1–15 μm and for TiO_2_ conjugate 1–25 μm.

## Conflict of interest

The authors declare no conflict of interest.

## Author contributions

GJ designed the project, served as senior and corresponding author and was academic supervisor for EB. EB shared the design planning, wrote the first draft of the manuscript, performed or participated in most experiments and participated in the final writing steps. JV provided expertise in designing and leading the peptide synthesis work and participated in the writing of the manuscript. SBL planned and performed MS experiments and participated in the writing of the manuscript.

## Supporting information


**Fig. S1.** (A) MS spectra of oxidized leupeptin with a peak at 443 *m/z* corresponding to the molecular ion [M + H]. (B) MS spectra of reduced leupeptin with a peak at 429 *m/z* corresponding to the molecular ion [M + H], confirming the identity of both products.
**Fig. S2.** Kinetic measurements of TiO_2_–peptide conjugate.
**Fig. S3.** Erosion well formation rate for the free Ahx–Phe–Leu–Arg–COOH and in its conjugated state with increasing [Ahx–Phe–Leu–Arg–COOH] as are marked accordingly: (●) no inhibitor, (■) 1 μm, (♢) 5 μm, (▲) 10 μm, (○) 15 μm, (▼)25 μm, (♦)50 μm.
**Table S1.** Extracted values from Fig. S2. Experiment setup: buffer 0.1 m NH_4_HCO_3_ (pH 7.52), [trypsin] (in Eppendorf) = 0.125 μm, 34 mg TiO_2_–Ahx–Phe–Leu–COOH (6.06 nmol mod. peptide/mg TiO_2_) in each reaction.
**Table S2.** The rates, given by the linear regression, are given for the gel experiment for free Ahx–Phe–Leu–Arg–COOH and its conjugated state to TiO_2_ and ZnO.

## Data Availability

For information about raw data, that is, direct instrumental output, contact the corresponding author.

## References

[1] Maeda K , Kawamura K , Hondo S , Aoyagi T , Takeuchi T and Umezawa H (1971) The structure and activity of leupeptins and related analogs. J Antibiotics 24, 402–404. 10.7164/antibiotics.24.402 4253693

[2] Aoyagi T , Miyata S , Nanbo M , Kojima F , Matsuzaki M , Ishizuka M , Takeuchi T and Umezawa H (1969) Biological activities of leupeptins. J Antibiot 22, 558–568. 10.7164/antibiotics.22.558 4243683

[3] Toyo‐oko T , Shimizu T and Masaki T (1978) Inhibition of proteolytic activity of calcium activated neutral protease by leupeptin and antipain. Biochem Biophys Res Commun 82, 484–491. 666855 10.1016/0006-291x(78)90900-2

[4] Tamura Y , Hirado M , Okamura K , Minato Y and Fujii S (1977) Synthetic inhibitors of trypsin, plasmin, kallikrein, thrombin, C1r, and C1 esterase. Biochem Biophys Acta 484, 417–422. 143965 10.1016/0005-2744(77)90097-3

[5] Hopgood MF , Clark MG and Ballard FJ (1977) Inhibition of protein degradation in isolated rat hepatocytes. Biochem J 164, 399–407. 880245 10.1042/bj1640399 PMC1164805

[6] Takahashi S , Murakami K and Miyake Y (1981) Purification and characterization of porcine kidney cathepsin‐B. J. Biochem 90, 1677–1684. 7334002 10.1093/oxfordjournals.jbchem.a133643

[7] Summaria L , Wohl RC , Boreisha IG and Robbins KC (1982) A virgin enzyme derived from human plasminogen. Specific cleavage of the arginyl‐560‐valyl peptide bond in the diisopropoxyphosphinyl virgin enzyme by plasminogen activators. Biochemistry 21, 2056–2059. 7201324 10.1021/bi00538a012

[8] Azaryan A and Galoyan A (1987) Human bovine brain cathepsin‐L and cathepsin‐H‐purification, physicochemical properties and specificity. Neurochem Res 12, 207–213. 3574596 10.1007/BF00979539

[9] Kondo S , Kawamura K , Iwanaga J , Hamada M , Aoyagi T , Maeda K , Takeuchi T and Umezawa H (1969) Isolation and characterization of leupeptins produced by Actinomycetes. Chem Pharm Bull 17, 1896–1901. 10.1248/cpb.17.1896 5347594

[10] Suda H , Aoyagi T , Hamada M , Takeuchi T and Umezawa H (1972) Antipain, a new protease inhibitor isolated from actinomycetes. J. Antibiot 25, 263–266. 10.7164/antibiotics.25.263 4559651

[11] Aoyagi T and Umezawa H (1975) Structures and activities of proteinase inhibitors of microbiological origin In Proteases and Biological Control (Reich E, Rifkin DB and Shaw E, eds), pp. 429–454. Cold Spring Harbor‐Laboratory, Cold Spring Harbor, Academic Press, New York, NY.

[12] Saino T , Someno T , Ishii S , Aoyagi T and Umezawa H (1988) Protease‐inhibitory activities of leupeptin analogues. J Antibiot 2, 220–225. 10.7164/antibiotics.41.220 2965694

[13] Suzukake K , Hori M , Tamemasa O and Umezawa H (1981) Purification and properties of an enzyme reducing acid to Leupeptin. Biochem Biophys Acta 661, 175–181. 7295735 10.1016/0005-2744(81)90001-2

[14] McConnell RM , Barnes GE , Hoyng CF and Gunn JM (1990) New leupeptin analogues: synthesis and inhibition data. J Med Chem 33, 86–93. 2136920 10.1021/jm00163a014

[15] Bellamkonda RV , Ranieri JP and Aebischer P (1995) Laminin oligopeptide derivatized agarose gels allow 3‐dimensional neurite extension in‐vitro. J Neurosci Res 41, 501–509. 7473881 10.1002/jnr.490410409

[16] Borkenhagen M , Clémence JF , Sigrist H and Aebischer P (1998) Three‐dimensional extracellular matrix engineering in the nervous system. J Biomed Mater Res 40, 392–400. 9570070 10.1002/(sici)1097-4636(19980603)40:3<392::aid-jbm8>3.0.co;2-c

[17] Kurinov IV and Harrison RW (1996) Two crystal structures of the leupeptin‐trypsin complex. Protein Sci 5, 752–758. 8845765 10.1002/pro.5560050420 PMC2143399

[18] Radisky ES , Lee JM , Lu CK and Koshland DE Jr (2006) Insights into the serine protease mechanism from atomic resolution structures of trypsin reaction intermediates. Proc Natl Acad Sci USA 103, 6835–6840. 16636277 10.1073/pnas.0601910103 PMC1458980

[19] Moulin A , Martinez J and Fehrentz J‐A (2007) Synthesis of peptide aldehydes. J Pept Sci 13, 1–15. 16998827 10.1002/psc.787

[20] Billinger E , Zuo S and Johansson G (2019) Characterization of serine protease inhibitor from solanum tuberosum conjugated to soluble dextran and particle carriers. ACS Omega 4, 18456–18464. 31720549 10.1021/acsomega.9b02815 PMC6844106

[21] Billinger E and Johansson G (2018) Kinetic studies of serine protease inhibitors in simple and rapid ‘active barrier’ model systems – diffusion through an inhibitor barrier. Anal Biochem 546, 43–49. 29408179 10.1016/j.ab.2018.01.022

[22] Kaiser E , Colescott RL , Bossinger CD and Cook PI (1970) Color test for detection of free terminal amino groups in the solid‐phase synthesis of peptides. Anal Biochem 34, 595–598. 5443684 10.1016/0003-2697(70)90146-6

[23] Fasman GD , ed (1976) Handbook of Biochemistry and Molecular Biology. Vol. 1, 3rd edn, Proteins, pp. 183–203. CRC Press, Cleveland, OH.

[24] Dixon M (1972) The graphical determination of Km and Ki. Biochem J 129, 197–202. 4630451 10.1042/bj1290197 PMC1174056

[25] Morrison JF (1969) Kinetics of the reversible inhibition of enzyme‐catalysed reactions by tight‐binding inhibitors. Biochim Biophys Acta 185, 269–286. 4980133 10.1016/0005-2744(69)90420-3

