# Supplementary material for: Inhibition properties of free and conjugated leupeptin analogues
Source: FEBS Open Bio. 2020 Nov 2;10(12):2605–15. doi: 10.1002/2211-5463.12994 (PMC7714073; doi:10.1002/2211-5463.12994)
Supplement: Supplementary file 1 — Fig. S1. (A) MS spectra of oxidized leupeptin with a peak at 443 m/z corresponding to the molecular ion [M + H]. (B) MS spectra of reduced leupeptin with a peak at 429 m/z corresponding to the molecular ion [M + H], confirming the identity of both products. Fig. S2. Kinetic measurements of TiO2–peptide conjugate. Fig. S3. Erosion well formation rate for the free Ahx–Phe–Leu–Arg–COOH and in its conjugated state with increasing [Ahx–Phe–Leu–Arg–COOH] as are marked accordingly: (●) no inhibitor, (■) 1 μm, (♢) 5 μm, (▲) 10 μm, (○) 15 μm, (▼)25 μm, (♦)50 μm. Table S1. Extracted values from Fig. S2. Experiment setup: buffer 0.1 m NH4HCO3 (pH 7.52), [trypsin] (in Eppendorf) = 0.125 μm, 34 mg TiO2–Ahx–Phe–Leu–COOH (6.06 nmol mod. peptide/mg TiO2) in each reaction. Table S2. The rates, given by the linear regression, are given for the gel experiment for free Ahx–Phe–Leu–Arg–COOH and its conjugated state to TiO2 and ZnO. [file FEB4-10-2605-s001.docx]

**SUPPLEMENT**

**S1a. Oxidized leupeptin**

*Figure S1a. MS spectra of oxidized leupeptin with a peak at 443 m/z corresponding to the molecular ion [M+H].*

**S1b. Reduced leupeptin**

*Figure S1b. MS spectra of reduced leupeptin with a peak at 429 m/z corresponding to the molecular ion [M+H] confirming the identity of both products.*


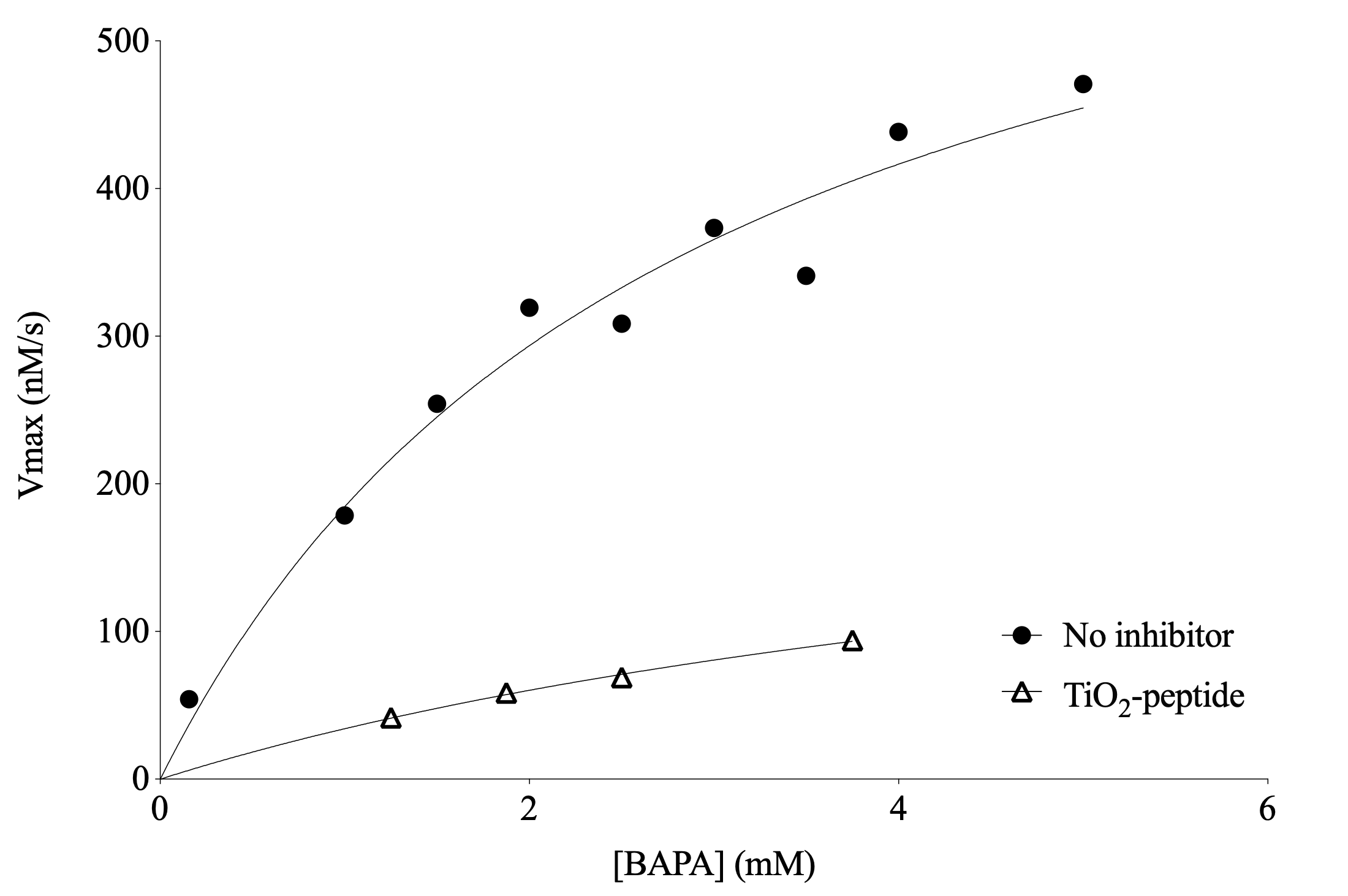

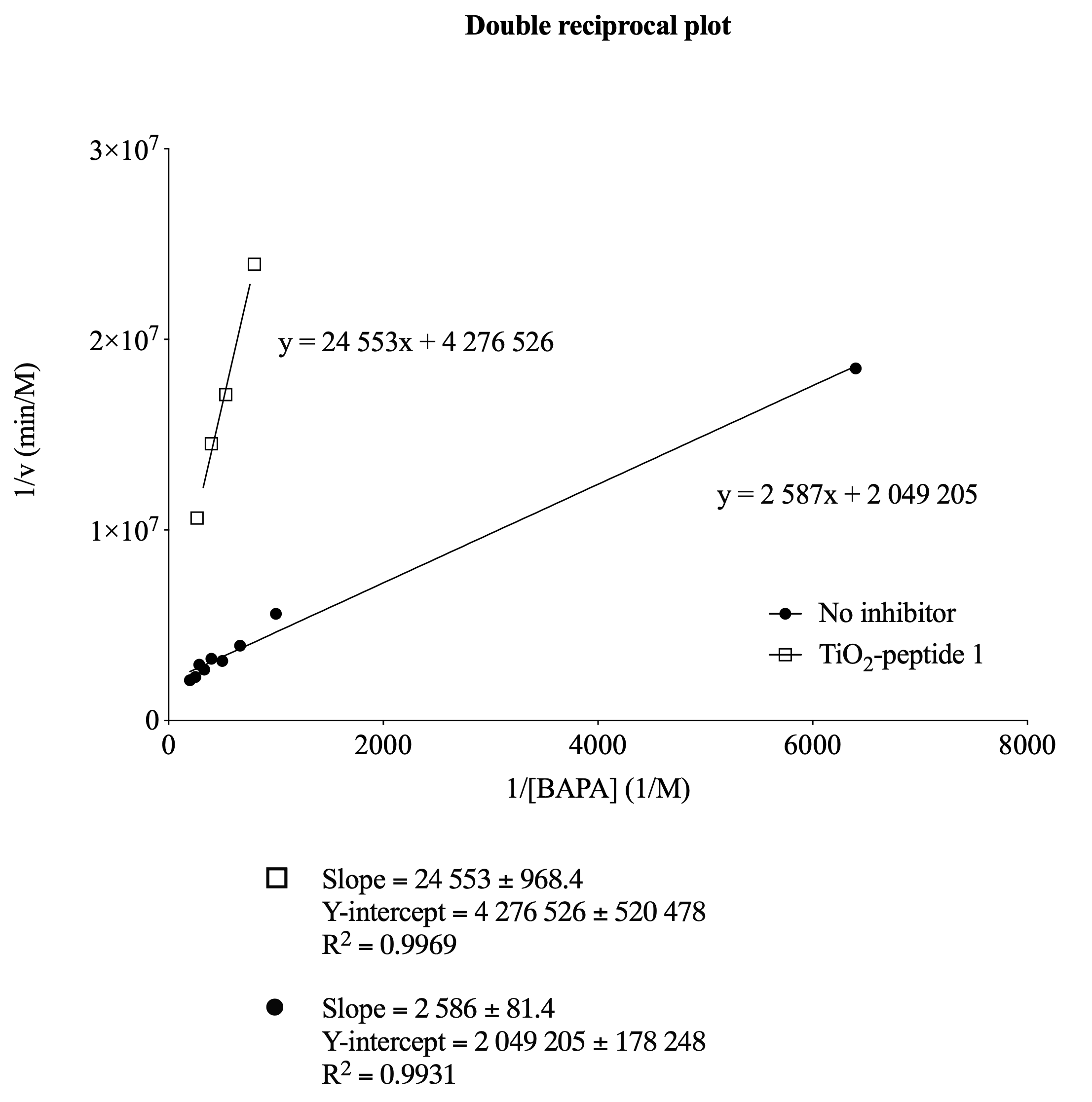


*Figure S2. Kinetic measurements of TiO_2_-peptide conjugate.*

**Table S1.** Extracted values from figure S2. Experiment setup: Buffer 0.1 M NH_4_HCO_3_ pH 7.52**,** [Trypsin] (in eppendorf) = 0.125 μM**,** 34 mg TiO_2_-Ahx-Phe-Leu-COOH

(6.06 nmol mod. peptide/mg TiO_2_) in each reaction.

|  | No inhibitor | TiO_2_-peptide 1 |
| --- | --- | --- |
| V_max_ | 720 ± 100 | 250 ± 30 |
| K_m_ | 2.9 ± 0.8 | 6.4 ± 1.2 |


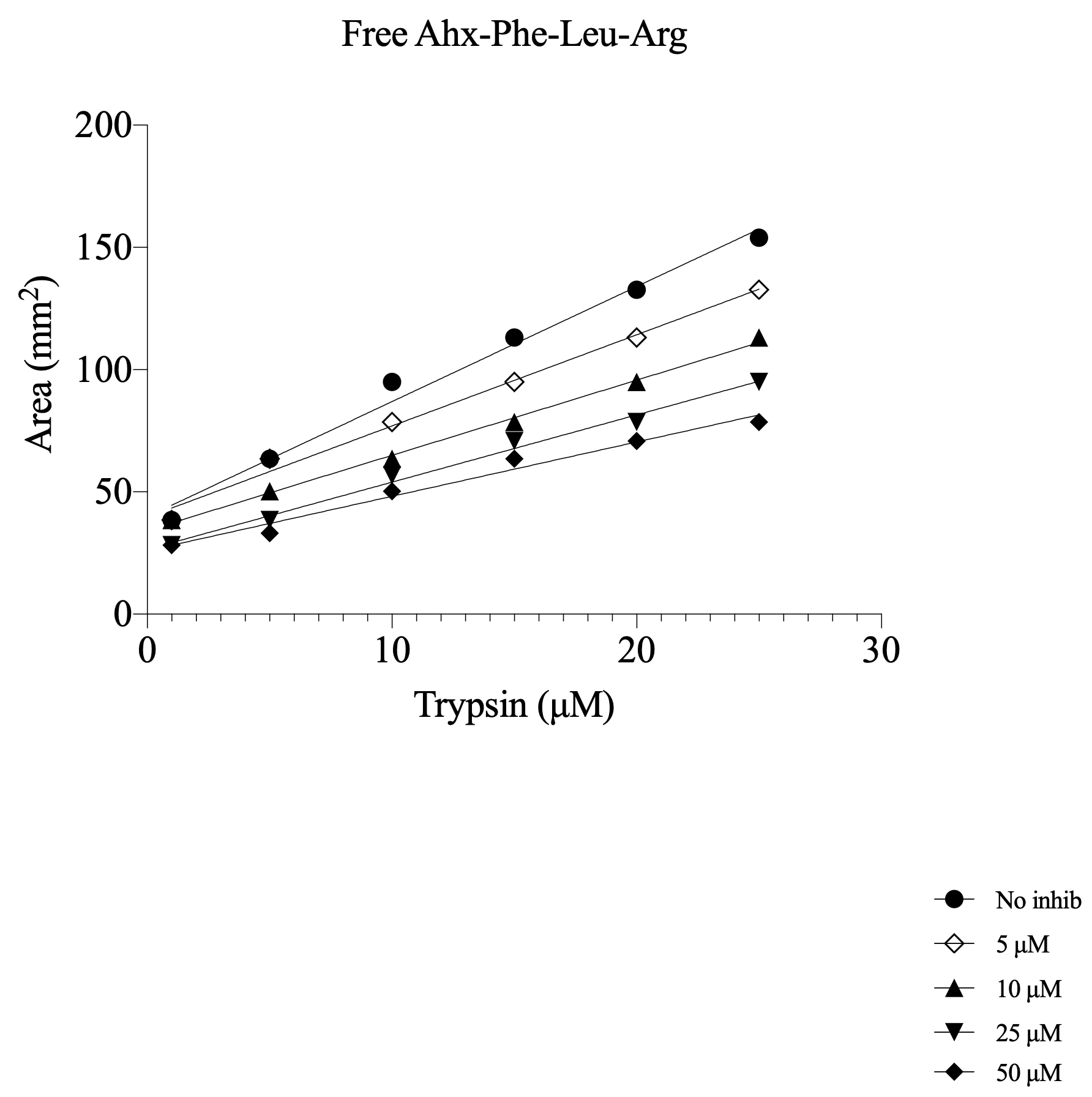

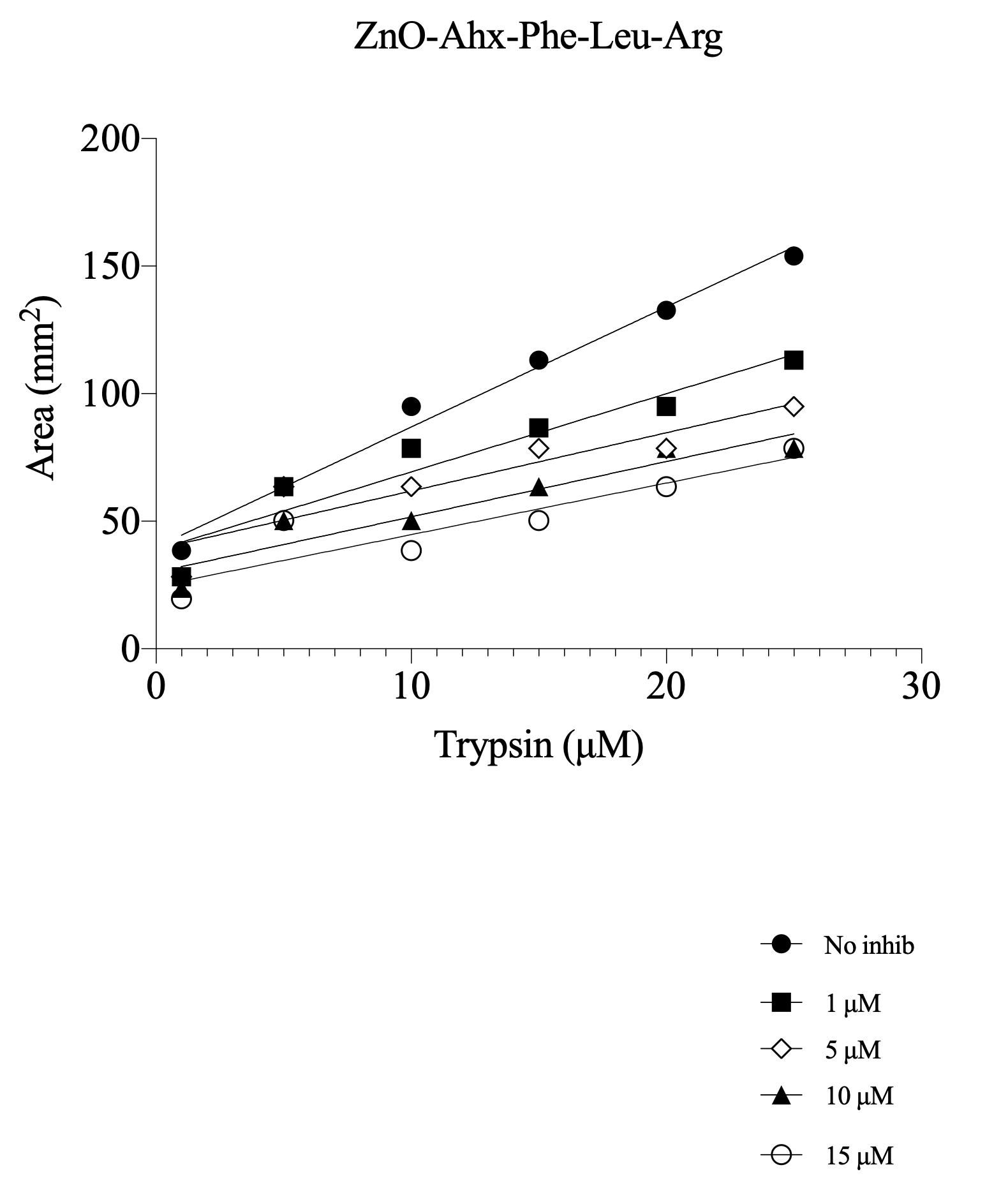

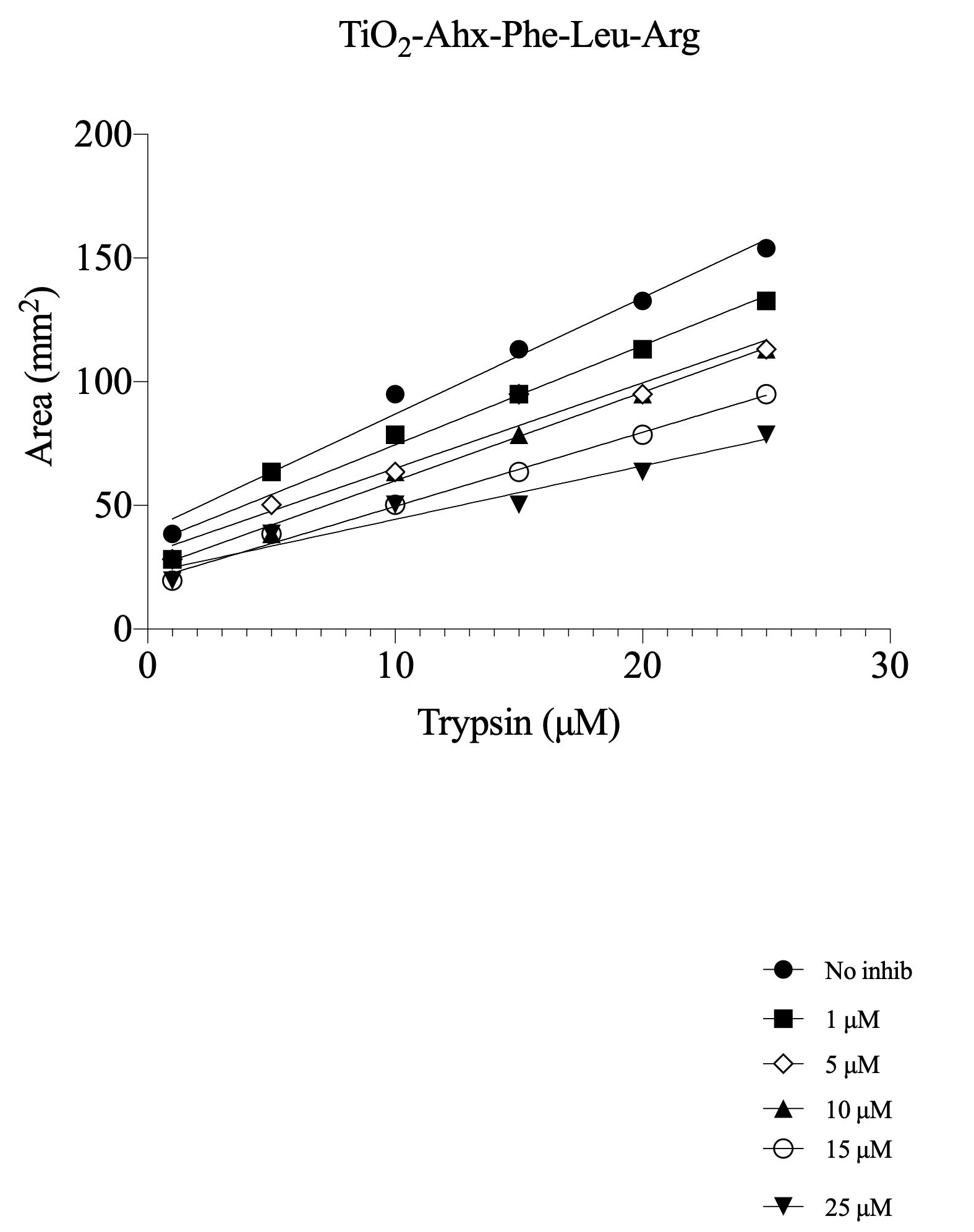


*Figure S3. Erosion well formation rate for the free Ahx-Phe-Leu-Arg-COOH and in its conjugated state with increasing [Ahx-Phe-Leu-Arg-COOH] as are marked accordingly:* (●) no inhibitor , (■) 1 μM, (◇) 5 μM, (▲) 10 μM, (○) 15 μM, (▼)25 μM, (◆)50 μM.

**Table S2.** The rates, given by the linear regression, are given below for the gel experiment for free Ahx-Phe-Leu-Arg-COOH and its conjugated state to TiO_2_ and ZnO.

|  | **Ahx-Phe-Leu-Arg** | | **ZnO-Ahx-Phe-Leu-Arg** | | **TiO_2_-Ahx-Phe-Leu-Arg** | |
| --- | --- | --- | --- | --- | --- | --- |
|  | **Rate  (mm^2^/ μM_trypsin_)** | **R^2^** | **Rate  (mm^2^/ μM_trypsin_)** | **R^2^** | **Rate  (mm^2^/ μM_trypsin_)** | **R^2^** |
| ● - No inhibitor | 4.7 ± 0.3 | 0.99 | 4.7 ± 0.3 | 0.99 | 4.7 ± 0.3 | 0.99 |
| ■ - 1 μM | nd | nd | 3.1 ± 0.5 | 0.91 | 4.0 ± 0.4 | 0.97 |
| ◇ - 5 μM | 3.7 ± 0.2 | 0.99 | 2.3 ± 0.50 | 0.84 | 3.5± 0.4 | 0.95 |
| ▲ - 10 μM | 3.1 ± 0.1 | 0.99 | 2.2 ± 0.4 | 0.90 | 3.6 ± 0.1 | 0.99 |
| ○ - 15 μM | Nd | nd | 2.0 ± 0.5 | 0.82 | 2.00 ± 0.1 | 0.99 |
| ▼ - 25 μM | 2.8 ± 0.1 | 0.99 | nd | nd | 2.2 ± 0.3 | 0.94 |
| ◆ - 50 μM | 2.2 ± 0.2 | 0.98 | nd | nd | nd | nd |
